# Antidiabetic Properties of Naringenin: A Citrus Fruit Polyphenol

**DOI:** 10.3390/biom9030099

**Published:** 2019-03-12

**Authors:** Danja J. Den Hartogh, Evangelia Tsiani

**Affiliations:** 1 Department of Health Sciences, Brock University, St. Catharines, ON L2S 3A1, Canada; dd11qv@brocku.ca; 2 Centre for Bone and Muscle Health, Brock University, St. Catharines, ON L2S 3A1, Canada

**Keywords:** insulin resistance, diabetes, naringenin, naringin, skeletal muscle, adipose, liver, pancreas

## Abstract

Type 2 diabetes mellitus (T2DM) is a metabolic disease characterized by insulin resistance and hyperglycemia and is associated with personal health and global economic burdens. Current strategies/approaches of insulin resistance and T2DM prevention and treatment are lacking in efficacy resulting in the need for new preventative and targeted therapies. In recent years, epidemiological studies have suggested that diets rich in vegetables and fruits are associated with health benefits including protection against insulin resistance and T2DM. Naringenin, a citrus flavanone, has been reported to have antioxidant, anti-inflammatory, hepatoprotective, nephroprotective, immunomodulatory and antidiabetic properties. The current review summarizes the existing in vitro and in vivo animal studies examining the anti-diabetic effects of naringenin.

## 1. Introduction

### 1.1. Glucose Homeostasis: Role of Insulin

Insulin is a protein hormone primarily involved in glucose and nutrient homeostasis. Insulin is produced by the β-cells of the pancreatic islets of Langerhans [1,2,3]. In response to elevated blood glucose levels, following a meal, the β-cells release insulin into the circulation to be transported to its target tissues, including skeletal muscle, adipocytes, and hepatocytes [1,2,3]. Insulin increases glucose uptake by skeletal muscle and adipose tissue, while it suppresses the endogenous production of glucose by the liver resulting in a reduction/restoration of blood glucose back to normal levels [1,2,3].

Insulin initiates its mechanism of action through binding to its receptor located on the plasma membrane of its target cells. Insulin binding to its receptor leads to increased receptor tyrosine kinase activity, phosphorylation of the insulin receptor substrate (IRS), and downstream activation of the lipid kinase phosphatidylinositol-3 kinase (PI3-K) and the serine/threonine kinase Akt/PKB [2,3,4]. In adipose and muscle cells, this leads to downstream glucose transporter (GLUT4) translocation from an intracellular compartment to plasma membrane and entry of glucose, while in liver cells, the result is suppression of glycogenolysis and gluconeogenesis and reduced endogenous glucose production [3]. Impairments in insulin signaling leads to insulin resistance, and type 2 diabetes mellitus (T2DM) [2,3,4].

Insulin resistance is a condition characterized by reduced responsiveness of target tissues to normal circulating levels of insulin [2,4,5]. Insulin resistance/T2DM is associated with inflammation, obesity, aging and a sedentary lifestyle and results in chronic elevations of plasma glucose levels, known as hyperglycemia, that can lead to long-term complications including macrovascular and microvascular damage, cardiovascular disease, retinopathy, neuropathy, and nephropathy [2,4,5,6,7,8,9]. Obesity is strongly linked to insulin resistance and excess plasma free-fatty acids (FFA) have been established to impair the ability of insulin to suppress hepatic glucose output and to stimulate glucose uptake by skeletal muscle [7,9]. Furthermore, strong evidence have established that chronic inflammation contributes to insulin resistance. Pro-inflammatory cytokines such as, tumor necrosis factor-α (TNF-α), reduce the insulin-stimulated tyrosine phosphorylation of the insulin receptor and IRS-1, impairing insulin action and inducing insulin resistance [10]. T2DM accounts for 90%–95% of all diabetes cases [2]. The global burden of diabetes continues to rise and resulted in 5 million deaths in 2017, in the 20+ age demographic, compared to 665,000 deaths in 1990 [11]. Additionally, it is estimated that approximately 451 million people, aged 18+ are living with diabetes worldwide [11]. Diabetic complications such as diabetic foot and diabetic neuropathy significantly increased by 47.1% and 62.6%, respectively, from 2001 to 2014 [12], and overall the rise of diabetes cases exerts a significant economic burden on the health care systems globally [13,14]. For example, an incidence predictive study on the cost of diabetes in Canada over 10 years has estimated 2.16 million new cases of diabetes occurring during this timeframe, which is accompanied by a health care costs of $15.36 billion due to acute hospitalizations and prescription medications [15]. 

Epidemiological studies have suggested that diets high in fruits and vegetables help regulate body weight and protect against chronic diseases such as cardiovascular disease, cancer, and diabetes [16,17,18]. However, the role the individual components of these foods play in disease prevention and treatment is difficult to determine. Specific components, known as polyphenols, have increasingly gained attention within the scientific community for their potential health benefits and preventive and therapeutic properties against chronic diseases [13,14,19,20,21,22]. 

Polyphenols have been established to have antioxidant properties [23] and possess a variety of other specific biological effects such as regulating enzymes and, therefore, may prevent diseases through mechanisms that are both dependent and independent of their antioxidant properties [24,25,26]. 

### 1.2. Naringenin

**Figure 1:**
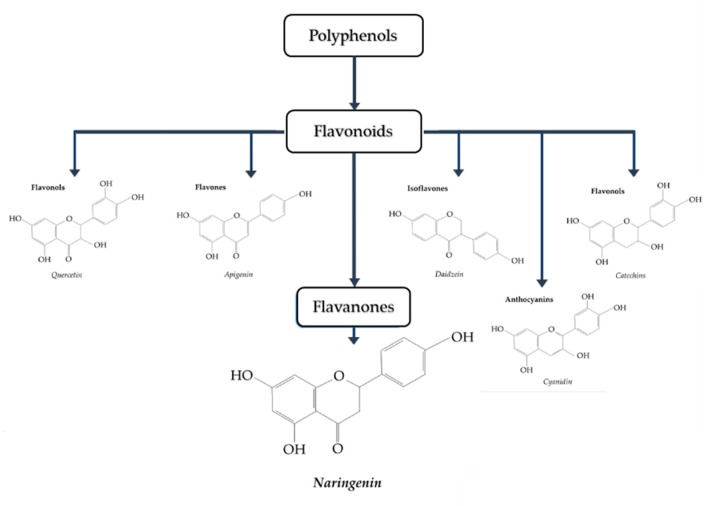
Classification of polyphenols. Naringenin belongs to the class known as flavanones [27].

Naringenin (4,5,7-trihydroxy-flavanone) (Figure 1 [27]) is a polyphenol belonging to flavonoids, the largest class of polyphenols with over 6000 identified sharing the common structure of 2 aromatic rings joined by a linear 3 carbon chain (C6-C3-C6) that forms an oxygenated heterocycle [28]. In naringenin, a flavanone subclass of flavonoids, this heterocycle contains a saturated 3-carbon chain and an oxygen atom at carbon 4 [28]. Naringenin is found mainly in citrus fruits, with considerably high levels found in grapefruit (43.5 mg/100 mL), lower levels found in orange juice (2.13 mg/100 mL), and much lower levels found in lemon juice (0.38 mg/100 mL) [29,30].

The formation of flavonoids, including naringenin, in plants/fruits is influenced by numerous factors such as plant genetics, environmental conditions (soil and light), germination, degree of ripeness, processing and storage [31]. Naringenin has been studied for its pharmacological effects, including antioxidant, anti-inflammatory, immunomodulatory, hepatoprotective, nephroprotective, neuroprotective, anti-cancer, anti-atherosclerotic, and anti-diabetic properties [32,33,34,35,36,37,38,39,40,41,42]. 

There are limited number of studies examining the bioavailability and pharmacokinetics of naringenin. Administration of grapefruit juice (250 mL) containing approximately 200 mg of naringenin in healthy individuals, resulted in peak plasma naringenin concentrations that ranged from 0.7 µM to 14.8 µM [43]. In another study, administration of 135 mg of naringenin resulted in peak plasma naringenin levels of 2009.51 ± 770.82 ng/mL [44]. Given that the molecular weight of naringenin is 272.257 g/mole, the plasma concentration of naringenin of 2 µg/mL corresponds to 7.34 µM. These two studies indicate that micromolar levels of naringenin can be reached in plasma after administration of grapefruit juice [43] or pure naringenin [44]. More studies should be performed to examine plasma naringenin levels and bioavailability in humans. Furthermore, there are no studies examining tissue distribution of naringenin in humans. Administration of 2.5 mg naringenin by gastric gavage in Sprague–Dawley rats resulted in the accumulation of naringenin in plasma, brain, liver, kidney, small intestine, large intestine, and feces. Approximately 42.11% of the initial naringenin was found in the urine, indicating increased clearance by the kidneys [45]. 

The current review is focused on the anti-diabetic effects of naringenin and all existing in vitro and in vivo animal and human studies are presented. A PubMed search was performed using the key words: naringenin, skeletal muscle, adipocyte, hepatocyte, β-cell, pancreas, streptozotocin (STZ), alloxan, high-fat diet, obesity and diabetes. These key words were searched in multiple different combinations to ensure that all existing in vitro and in vivo animal and human studies were included. The studies are presented chronologically, and in addition to the text, are organized and presented in a table format to allow for easier access of the information by the reader.

## 2. Anti-Diabetic Effects of Naringenin

### 2.1. Effects of Naringenin: In Vitro Skeletal Muscle Cell Studies

Zygmunt, et al. (2010) using L6 muscle cells found an increase in glucose uptake by naringenin (75 µM, 2 h) treatment [46]. In addition, naringenin significantly increased 5′ AMP-activated protein kinase (AMPK) phosphorylation/activation, and AMPK silencing using small interference RNA abolished the naringenin-stimulated glucose uptake (Table 1) [46].

Using primary porcine myotubes Bhattacharya et al., (2013) found that exposure to the *Sambucus nigra* flower (elderflower) and its major polyphenol, naringenin (0.1–10 µM), significantly increased glucose uptake [47]. In addition, exposure of the myotubes to elderflower extract (100–500 µg/mL), containing significant levels of naringenin (representing 23% of total phenolic compounds analyzed), reduced the formation of intracellular reactive oxygen species (ROS) (Table 1) [47]. Increased ROS formation leads to increased oxidative DNA damage, protein modification, and is associated with insulin resistance [49] while antioxidants such as N-acetylcysteine (NAC) counteract insulin resistance [50]. These data [47] indicate that naringenin may act as an antioxidant to increase muscle glucose uptake.

In L6 myotubes rendered insulin resistant by exposure to the free-fatty acid (FFA) palmitate (750 µM) treatment with naringenin (50 µM and 75 µM) for 16 h abrogated the effects of palmitate by significantly restoring the insulin-stimulated glucose uptake and GLUT4 translocation [48]. Additionally, naringenin increased the phosphorylation of AMPK and the levels of SIRT1 and peroxisome proliferator-activated receptor gamma coactivator 1-alpha (PGC-1α) (Table 1) [48]. 

Overall, these studies (Table 1) indicate that in skeletal muscle cells naringenin has the potential to activate AMPK, increase glucose uptake and counteract the palmitate-induced insulin resistance. Some of these effects of naringenin may be related to its antioxidant properties. 

### 2.2. Effects of Naringenin: In Vitro Adipocyte Studies

Treatment of 3T3-L1 pre-adipocytes with naringenin (5–100 µM) for 48 h significantly decreased proliferation in a dose-dependent manner (Table 2) [51]. This was accompanied with a significant increase in lactic acid dehydrogenase (LDH) release without an effect on triglyceride accumulation or adipogenesis gene expression (*PPAR-γ* and *STAT3*) during adipocyte differentiation [51].

Elevated levels of the pro-inflammatory cytokine TNF-α is associated with insulin resistance. Treatment of 3T3-L1 adipocytes with naringenin (100 µM) for 30 min significantly reduced the TNF-α-induced FFA secretion [52]. Treatment with naringenin resulted in increased IκB-α cytosolic levels and decreased p-ERK protein levels. In addition, naringenin treatment significantly increased the antilipolytic gene (perilipin and *PDE3B*) mRNA levels (Table 2) [52]. 

A study by Claussnitzer et al., (2011) using 3T3-L1 adipocytes and mature human adipocytes found that treatment with naringenin (20 µM) significantly reduced the insulin-stimulated glucose transport and reduced the insulin-stimulated GLUT4 translocation (Table 2) [53]. 

Toll-like receptors (TLRs) are involved in the obesity-induced inflammation of adipose tissue and contribute to insulin resistance and T2DM. In 3T3-L1 differentiating adipocytes, TLR2 expression was significantly inhibited in a dose-dependent manner by naringenin (10–100 µM) treatment (Table 2) [54]. This was accompanied by a significant decrease in inflammatory mediators, TNF-α and monocyte chemoattractant protein (MCP)-1 levels. In addition, naringenin suppressed the TNF-α-induced and macrophage co-culture-induced TLR2 adipocyte expression. These effects were associated with the inhibition of the NF-κB and JNK signaling cascades by naringenin [54]. 

In a study by Richard et al., (2013), 3T3-L1 differentiating and differentiated adipocytes treated with naringenin (25 µM) resulted in impaired mature adipocyte function and inhibition of adipogenesis [55]. Naringenin treatment (0–120 h) of immature 3T3-L1 cells resulted in the inhibition of lipid accumulation and decreased protein expression of aP2, PPARγ, STAT5A and adiponectin, all involved in adipogenesis. In mature differentiated 3T3-L1 adipocytes naringenin treatment prevented the insulin-induced glucose uptake, reduced tyrosine phosphorylation of IRS-1 and reduced adiponectin levels, indicating induction of insulin resistance (Table 2) [55]. These two studies [53,55] show an effect of naringenin to counteract insulin action in adipocytes in vitro, suggesting a potential negative effect on glucose homeostasis in vivo. 

On the other hand, in a recent study of human white adipocytes, treatment with naringenin (8 µM) for 7 to 14 days resulted in increased thermogenesis [56]. Basal, adenosine triphosphate (ATP)-linked and maximal and reserve oxygen consumption rate was increased. Naringenin treatment increased mRNA levels of UCP1, ATGL, CPT1β, PGC-1α and PGC-1β, all involved in fat oxidation. In addition, GLUT4, adiponectin and CREBpβ mRNA levels were increased with naringenin treatment [56]. This study indicates that naringenin increased energy expenditure, thermogenesis and insulin sensitivity in human adipocytes (Table 2). From all the above 6 studies, performed using adipocytes in culture, the evidence indicate that treatment with naringenin inhibits adipocyte proliferation [51] indicating a potential to inhibit adipose tissue expansion seen in obesity. The reduction in the levels of the inflammatory mediators TNF-α and MCP-1 [54], the reduced action of TNF-α [52], together with the reduced TLRs levels [54] seen with naringenin treatment point to its anti-inflammatory properties in adipocytes. The increased adipocyte thermogenesis and increased energy expenditure by naringenin treatment [56] is novel and indicates a potential to convert white adipose to brown adipose tissue which is associated with increased insulin sensitivity and anti-diabetic potential [57].

As mentioned above, two studies [53,55] show an effect of naringenin to counteract insulin action in adipocytes and point to the need for more studies to clarify the effects of naringenin in adipose tissue.

### 2.3. Effects of Naringenin: In Vitro Hepatocyte Studies

Apolipoprotein B (ApoB) is a structural protein that is an integral component of very-low density lipoprotein (VLDL) and is sequestered in the liver postprandially. However, insulin resistance reduces this sequestering, allowing ApoB to be readily secreted from hepatocytes and drive the formation of VLDLs. HepG2 human hepatoma cells treated with naringenin (10–200 µM) for 24 h had significantly reduced ApoB accumulation and cellular cholesteryl ester mass (Table 3) [58]. In addition, naringenin treatment significantly reduced acetyl-CoA acetyltransferase 2 (ACAT2) mRNA levels and microsomal triglyceride transfer protein (MTP) levels and activity. Naringenin treatment significantly increased LDL receptor mRNA levels and increased LDL uptake and degradation [58].

Treatment of HepG2 cells with naringenin (200 µM) for 7 h resulted in a significant reduction of ApoB secretion [59]. This was accompanied with an increase in HepG2 cell PI3K activity, without an effect on IRS-1 tyrosine phosphorylation. In addition, naringenin treatment increased sterol regulatory element-binding protein-1 (SREBP-1) and low-density lipoprotein receptor (LDLr) expression in a PI3K-dependent manner (Table 3) [59]. 

In a follow up study [60] by the same group [59] it was found that the inhibition of ApoB secretion from HepG2 cells by insulin was amplified by naringenin treatment clearly indicating insulin sensitizing effects of naringenin [60]. Importantly, insulin receptor or IRS-1 tyrosine phosphorylation was not affected, suggesting an effect of naringenin that is independent of the insulin signaling cascade (Table 3) [60]. 

Exposure of hepatoma (Fao) cells to naringenin for 6 h resulted in dose-dependent suppression of glucose production (Table 3) [61]. Additionally, naringenin did not increase the phosphorylation/activation of Akt (S473) or Akt (T308) indicating that naringenin’s effects are independent of Akt. Naringenin treatment also significantly decreased cellular ATP levels, while not influencing cytotoxicity [61]. 

Treatment of Huh7 hepatocytes with naringenin (200 µM) for 24 h resulted in reduced triglyceride production [62]. Additionally, naringenin treatment increased fatty acid oxidation, and the mRNA levels of genes involved in fatty acid oxidation (CYP4A11, ACOX, UCP1, ApoAI and PGC1α). Naringenin treatment inhibited the activation of the liver X receptor-α (LXRα) response element in human hepatocytes through the inhibition of the binding of Trap220/Drip-2 co-activator peptide to the LXRα-ligand binding domain (LBD). The interaction of Trap220/Drip-2 to the LXRα-LBD is involved in the transcriptional regulation of genes that control cholesterol efflux and fatty acid biosynthesis. Peroxisome proliferator response element (PPRE) activity was also increased dose-dependently following treatment with naringenin (Table 3) [62]. 

In the study by Constantin, et al., (2014), exposure of rat hepatocytes to naringenin (300 µM) resulted in a significant reduction of the lactate (2 mM) and pyruvate (0.2 mM)-induced gluconeogenesis. The reduction in hepatocyte glucose production was associated with a reduction of pyruvate carboxylation and an inhibition of pyruvate transport into the hepatocyte mitochondria (Table 3) [63]. 

Overall, these studies (Table 3) indicate that in hepatocytes naringenin reduces ApoB secretion, triglyceride production and gluconeogenesis and these effects may lead to attenuation of hyperglycemia, hyperlipidemia and insulin resistance in vivo. 

### 2.4. Effects of Naringenin: In Vitro Beta Cell Studies

Only one study exists examining the effect of naringenin on pancreatic β-cells [64]. Treatment of rat pancreatic INS-1E cells with naringenin (100–1000 µM) enhanced the glucose-stimulated insulin secretion and increased the expression of several β-cell genes *including Glut2, Gck, Ins1, Ins2, Beta2, Pdx1, Akt1, Akt2, Irs1, Bcl2*, and *Hsp70/90*. Naringenin treatment decreased pro-apoptotic marker mRNA levels, including *Casp3, Bax* and *Acc1* (Table 4) [64]. Overall, this study suggests an effect of naringenin in β-cells to increase glucose sensitivity and protect β-cells from apoptosis. 

### 2.5. Evidence of Anti-Diabetic Effects of Naringenin: In Vivo Animal Studies

#### 2.5.1. Streptozotocin (STZ)-Induced Diabetes Model

The effects of naringenin were examined in a few studies using streptozotocin (STZ)-induced animal models. T2DM was induced in vivo in neonatal Wistar rats by an intraperitoneal (i.p) injection of STZ (100 mg/kg) in the study by Li, et al., (2006) followed 21 days later by intestinal brush border membrane vesicle (BBMV) and renal cortical BBMV in vitro tissue isolation (Table 5) [65]. Naringenin treatment dose-dependently reduced intestinal sleeve and kidney glucose uptake. Additionally, naringenin dose-dependently decreased Na+-dependent glucose uptake activities in the diabetic rat kidney BBMVs [65]. New Zealand White rabbits were also used in the above study [65], mimicking the results in the neonatal Wistar rats. Naringenin treatment dose-dependently reduced glucose uptake in rabbit intestinal BBMVs [65]. These studies provide evidence of an effect of naringenin to inhibit intestinal glucose absorption as well as renal glucose reabsorption, contributing to an attenuation of diabetic hyperglycemia (Table 5).

Naringenin treatment (50 mg/kg b.w./day) for 5 days of STZ and nicotinamide-induced diabetic Wistar rats resulted in reduced blood glucose, total cholesterol and triglyceride levels [66]. Additionally, serum high-density lipoprotein (HDL) levels were increased by naringenin treatment (Table 5) [66].

Naringin (30 mg/kg b.w.) and vitamin C (25 mg/kg b.w.) co-administered for 21 days in Wistar rats resulted in the attenuation of the hyperglycemia and oxidative stress induced by the single i.p. injection of STZ (45 mg/kg b.w.) (Table 5) [67]. Naringin treatment significantly reduced serum glucose levels and increased serum insulin levels, attenuating the diabetic phenotype. Additionally, treatment with naringin increased liver and kidney hexokinase activity and reduced liver and kidney glucose-6-phosphatase (G6Pase) and fructose-1,6-bisphosphatase (F16BPase) activities. Hexose, hexosamine, fucose and sialic acid levels were decreased in the plasma, kidney and liver of diabetic rats with naringin treatment [67]. Therefore, the combined treatment of naringin and vitamin C provided antihyperglycemic and antioxidant effects attenuating the diabetic phenotype. 

Administration of naringenin (1% and 2% of diet) for 10 weeks in STZ-induced diabetic mice resulted in significantly reduced blood glucose and urea levels, while serum insulin levels were increased (Table 5) [68]. Renal TNF-a, interleukin (IL)-1B, IL-6 and MCP-1 mRNA and protein levels were reduced. Furthermore, kidney type IV collagen, fibronectin and transforming growth factor-β1, mRNA and protein levels were reduced. This was accompanied with the suppression of kidney NF-κB p65 mRNA and protein levels [68].

In the study by Sharma, et al., (2011), Wistar albino male rats fed a high-fat diet (55% fat, 2% cholesterol) and administered a single i.p. injection of STZ (40 mg/kg b.w.) to induce diabetes, were treated with naringin (25, 50 and 100 mg/kg b.w./day) for 28 days (Table 5) [69]. Naringin decreased serum glucose and insulin levels in a dose-dependent manner. Overall lipid profile was improved with naringin treatment with serum triglyceride, triacylglycerol, LDL cholesterol and non-esterified fatty acid levels reduced, and HDL-cholesterol levels increased [69]. Additionally, treatment with naringin prevented pancreatic islet cell destruction and preserved β-cell insulin granule content. Liver and kidney PPARγ protein levels, and liver HSP-72 and HSP-27 protein levels were increased with naringin treatment. Pro-inflammatory cytokine NF-κB p 65 protein levels in the pancreas, liver and kidney and serum TNF-α, IL-6 and CBP levels were significantly reduced with naringin. Superoxide dismutase (SOD) and glutathione peroxidase (GSH-Px) activities were increased and thiobarbituric acid reactive substances (TBARS) levels were reduced in the kidney, pancreas and liver with naringin treatment [69].

Diabetes was induced in albino Wistar rats by intraperitoneal injections of STZ (50 mg/kg b.w.) and nicotinamide (110 mg/kg b.w.) in a study by Annadurai, et al., (2012), followed by oral administration of naringenin (50 mg/kg b.w./day) for 21 days [70]. Treatment with naringenin significantly decreased fasting serum glucose and glycosylated hemoglobin HbA1c levels, while insulin levels were increased. Additionally, naringenin significantly increased the activities of pancreatic antioxidant enzymes (SOD, catalase, GSH-Px and glutathione-S-transferase) and non-enzymatic antioxidants (glutathione (GSH), vitamin C and vitamin E) plasma levels (Table 5) [70]. 

Administration of naringin (50 mg/kg b.w.) for 4 weeks in male albino high-fat fed/STZ-induced diabetic rats reversed the diabetic phenotype [71]. Naringin treatment significantly decreased serum glucose and HbA1c levels, while insulin levels were increased. Serum and hepatic lipid peroxide and nitric oxide levels were reduced in diabetic rats treated with naringin. Serum GSH levels were significantly increased with naringin treatment. Pro-inflammatory cytokines, TNF-α and IL-6 serum levels were significantly increased in diabetic animals and naringin treatment normalized (reduced) to levels similar to control (Table 5) [71]. 

Administration of naringenin (25 mg/kg p.o.) in high-fat diet fed/STZ-induced diabetic rats reduced the maltose- and sucrose-loaded glycemic response by 49.72% and 49.96%, respectively (Table 5) [72]. Additionally, naringenin significantly inhibited the activity of intestinal α-glucosidase resulting in decreased carbohydrate absorption and reduced postprandial blood glucose levels [72]. 

Treatment of STZ-induced diabetic rats with naringenin (50 and 100 mg/kg) significantly decreased serum glucose levels, and increased serum SOD antioxidant activity [73]. In addition, treatment with naringenin protected against diabetic hyperalgesia and tactile allodynia (Table 5) [73]. 

Administration of naringenin (50 mg/kg b.w.) for 30 days in male Wistar STZ-induced diabetic rats decreased blood glucose levels and decreased hepatocyte ROS and lipid peroxidation [74]. These anti-hyperglycemic effects were accompanied by an increase in hepatocyte mitochondrial membrane potential and decreased hepatocyte mRNA and protein levels of apoptosis regulators Bax and Bcl-2 [74]. Overall, treatment with naringenin provided hepatoprotection from STZ-induced diabetes.

Naringenin treatment (25 and 50 mg/kg b.w./day) for 5 weeks in STZ-induced diabetic rats reduced serum glucose levels and increased insulin levels [75]. This was accompanied with a dose dependent decrease in serum pro-inflammatory cytokine levels TNF-α, IL-1β, and IL-6 with naringenin treatment. Sciatic nerve insulin growth factor (IGF) and nerve growth factor (NGF) levels were significantly increased in naringenin treated-diabetic rats compared to control diabetic rats. Additionally, SOD and catalase activities were significantly increased, while TBARs and GSH levels were decreased following naringenin treatment. This resulted in an attenuation of the diabetic phenotype with decreased sciatic nerve axonal degenerative histological changes (Table 5) [75].

Intragastric administration of naringenin (50 or 100 mg/kg/day) for 6 weeks in STZ-induced diabetic rats significantly decreased blood glucose levels [76]. In addition, lipid, malonaldehyde and ICAM-1 serum levels were decreased with naringenin treatment and overall, a significant alleviation of the diabetic phenotype was seen (Table 5) [76]. 

Treatment of STZ and high-fat/high-sucrose fed-induced diabetic rats with naringenin (50 mg/kg b.w./day) for 6 weeks resulted in decreased serum glucose and urinary protein levels, indicating better kidney function (Table 5) [77]. This was accompanied with increased creatinine clearance and reduced glomerular area, reflecting improved renal filtration function. Naringenin reduced collagen (Col4) and fibronectin mRNA and protein levels indicating less deposition of extracellular matrix proteins in the kidneys. Furthermore, kidney TGF-β1, TGFBR1, smad2 and smad7 protein levels were decreased following naringenin treatment, while let-7a levels were increased. These results indicate naringenin acts to alleviate nephropathy in STZ-induced diabetic rats [77].

Administration of naringenin (5–10 mg/kg b.w.) for 10 weeks in STZ-induced diabetic rats significantly decreased serum glucose levels, total cholesterol, triglyceride, LDL, VLDL, creatine, albumin and urea levels (Table 5) [78]. Kidney tissue malondialdehyde levels were also reduced following naringenin treatment. Treatment with naringenin increased SOD, catalase and GSH enzyme activities in the diabetic kidney. Naringenin treatment improved kidney tissue histology through reduced apoptotic activity. Additionally, a significant decrease in renal tissue IL-1 expression occurred with naringenin treatment [78].

Oral administration of naringenin (50 mg/kg b.w./day) for 5 weeks in STZ (65 mg/kg b.w.)-induced diabetic rats significantly reduced serum TBARs levels and increased GSH levels [79]. Additionally, naringenin increased the levels of neuroprotective factors (BDNF), tropomyosin related kinase B (TrkB) and synaptophysin in the diabetic retina. Anti-apoptotic Bcl-2 protein expression was improved following naringenin treatment, while pro-apoptotic Bax and caspase-3 protein expression was reduced. This was accompanied with the overall improvement of the diabetic phenotype, with decreased fasting blood glucose levels and increased insulin levels with naringenin treatment (Table 5) [79]. 

Oral administration of naringenin (100 mg/kg b.w./day) for 4 weeks in nicotineamide (120 mg/kg b.w.)/STZ (50 mg/kg b.w.)-induced diabetic rats alleviated the diabetic phenotype via insulinotropic effects and insulin improving action [80]. Naringenin administration (100 mg/kg b. w./day for 4 weeks) restored the lowered insulin and C-peptide serum levels and liver glycogen content (Table 5). The serum lipid profile was improved, HDL were increased while, total cholesterol, triglyceride, LDL, and FFAs levels were reduced and comparable to levels seen in healthy non-diabetic animals. The activities of liver G6Pase and glycogen phosphorylase were decreased with naringenin treatment. GLUT4, insulin receptor β subunit and adiponectin mRNA levels were enhanced in adipose tissue following naringenin treatment [80]. 

Administration of naringenin (100 mg/kg/day) for 15 days in STZ-induced diabetic rats significantly reduced blood glucose levels [81]. Naringenin treatment restored body weight and lipid serum levels in diabetic animals to levels similar to those found in the control non-diabetic group. Naringenin treatment normalized oxidative stress biomarkers in the liver and pancreas and increased PPARγ and GLUT4 mRNA and protein levels in adipose tissue (Table 5) [81]. 

Overall, these studies (Table 5) indicate that naringenin administration in STZ-induced diabetic animals resulted in restoration of blood glucose and lipid levels, increased GLUT4 translocation in adipose tissue, and reduced diabetic nephropathy. In addition, naringenin administration resulted in anti-inflammatory and antioxidant properties.

#### 2.5.2. Alloxan-Induced Diabetes Model

Treatment of alloxan-induced diabetic mice with naringenin (75 mg/kg b.w.) for 7 days, resulted in a significant increase in body weight and an overall improvement of cellular blood component levels compared to control diabetic mice [82]. Hematological and immunological blood parameters, including macrophage, leukocyte, erythrocyte, hemoglobin, hematocrit, and platelets levels were all increased following treatment with naringenin. Additionally, cholesterol levels in the naringenin-treated group were significantly reduced when compared to the control diabetic mice (Table 6) [82]. Overall, the survival of diabetic mice was significantly improved by naringenin treatment. 

In a study by Sirovina et al., (2016) oral administration of naringenin (50 mg/kg/day) for 2 days to alloxan-induced diabetic mice reduced lipid peroxidation levels in the liver and kidney tissue (Table 6) [83]. Additionally, naringenin treatment reduced the number of vacuolated liver cells and the degree of vacuolisation [83]. 

#### 2.5.3. Genetic Diabetes Model

*Db*/*db* mice, a commonly used genetic model of type 2 diabetes, treated for 5 weeks with naringin (0.2 g/kg diet) had reduced blood glucose and lipid levels (Table 7) [84]. Naringin significantly lowered blood glucose, triglyceride and total cholesterol levels. Hepatic lipogenic enzymes, fatty acid synthase (FAS), G6Pase and phosphatidate phosphatase (PAP) activities were significantly reduced with naringin supplementation. Additionally, hepatic fatty acid β-oxidation was reduced by 28% in mice supplemented with naringin. Plasma PON activity, which is associated with increased hydrolyzed lipid peroxide in lipoproteins was significantly elevated with naringin, suggesting antioxidant effects. The expression of hepatic glucose regulating enzymes were influenced by naringin treatment, with glucokinase mRNA significantly enhanced, while G6Pase and phosphoenolpyruvate carboxykinase mRNA levels reduced. Naringin supplementation significantly reduced hepatic GLUT2, and increased adipocyte GLUT4 levels [84].

Ldlr^−/−^ mice supplemented a diet with naringenin (3% wt/wt) for 8 weeks had significantly reduced fasting plasma glucose, insulin and cholesterol levels [85]. Energy expenditure was increased only during the light cycle, but not the dark cycle indicating increased expenditure during mice sleeping hours. This was accompanied with increased respiratory exchange ratio (RER) during the dark cycle, indicating that it is metabolically driven. Naringenin supplementation reduced hepatic triglyceride content and increased plasma β-hydroxybutyrate levels. Additionally, hepatic *Pgc1a*, *Cpt1a* and *Pnpla2* mRNA levels and fatty acid oxidation were increased with naringenin supplementation (Table 7) [85]. 

#### 2.5.4. Diet-Induced Diabetes Model

Apart from the studies using STZ- and alloxan-induced diabetic animal models, several studies exist using diet-induced diabetic animal models (Table 8). Supplementation of naringin (0.05%) for 8 weeks in male rabbits fed a high-cholesterol (0.5% dietary intake) diet resulted in decreased plasma LDL and cholesterol levels and increased HDL levels [86]. In addition, hepatic 3-hydroxy-3-methylglutaryl CoA reductase activity was increased, and acyl-CoA acyltransferase activity was reduced with naringin supplementation. Naringin supplementation significantly increased total fecal sterol content indicating increased intestinal cholesterol absorption [86].

Administration of high-fat fed Ldlr^−/−^ mice with naringenin (1–3% wt/wt) for 4 weeks corrected the excess VLDL production, reduced hepatic steatosis and attenuated dyslipidemia [87]. Importantly, these improvements were seen with naringenin administration without an effect on caloric intake or fat absorption [87]. Naringenin treatment significantly increased hepatic fatty acid oxidation, liver Pgc1α, Cpt1α and Aco mRNA levels and mitochondrial DNA content. In addition, naringenin administration reduced hepatic cholesterol and cholesterol ester synthesis, prevented muscle and liver lipogenesis and muscle triglyceride accumulation and reduced plasma glucose and insulin levels (Table 8) [87].

Naringenin treatment (3% wt/wt) of high-fat fed Ldlr^−/−^ mice for 6 months resulted in decreased fasting plasma triglyceride and cholesterol levels [38]. In addition, aortic plaque deposits and atherosclerosis in the aortic arch and abdomen was reduced with naringenin treatment. Liver triglyceride and cholesteryl ester mass was reduced by 80% indicating reduced hepatic steatosis with naringenin treatment (Table 8) [38].

Administration of naringenin (50 mg/kg b.w.) for 45 days in Wistar rats fed a high-fructose diet (60 g/100 g) decreased plasma glucose, insulin, triglyceride and free fatty acid levels [43]. Treatment with naringenin restored liver hexokinase, pyruvate kinase, G6Pase and F16BPase activities to levels comparable to levels seen in healthy non-diabetic animals. In addition, administration of naringenin improved insulin sensitivity and enhanced liver protein tyrosine kinase (PTK), while reduced protein tyrosine phosphatase (PTP) activity (Table 8) [43].

Treatment of high-fat/high-sucrose fed rats with naringenin (0.003%, 0.006% and 0.012% of diet) for 6 weeks reduced the total plasma and liver triglyceride and cholesterol levels (Table 8) [88]. In addition, naringenin treatment decreased adiposity and triglyceride content in adipose tissue. Liver PPARα, CPT-1 and UCP2 protein levels were increased with naringenin [88].

C57BL/6 mice fed a high-fat diet (37.1% fat, 42.2% carbohydrate and 20.5% protein) for 20 weeks resulted in the development of obesity, dyslipidemia, liver dysfunction and insulin resistance [89]. Importantly, treatment with naringin (0.2 g/kg diet) for 20 weeks attenuated these changes (Table 8). Naringin treatment increased hepatic fatty acid oxidation and increased AMPK phosphorylation/activation [89]. Furthermore, treatment of hepatocytes, isolated from C57BL/6 mice fed a high-fat diet, with naringin resulted in increased p-AMPKα and p-IRS1 protein levels (Table 8) [89]. 

Treatment of high-fat fed C57BL/6J mice with naringenin (0.5%–3% of dietary intake) for 4 months significantly reduced blood glucose levels, and increased insulin levels [54]. In addition, naringenin treatment decreased TNF-α, MCP-1 and TLR2 expression in adipose tissue (Table 8) [54]. 

In a study by Alam, et al. (2013) administration of naringin (100 mg/kg/day) for 16 weeks in high-fat/high-cholesterol fed Wistar rats resulted in improved glucose tolerance, with decreased serum glucose, insulin, cholesterol, triglyceride and non-esterified fatty acid (NEFA) concentrations (Table 8) [90]. Liver mitochondrial function was also improved with naringenin treatment, resulting in reduced inflammatory cell infiltration, collagen deposition, plasma aspartate transaminase (AST) and alanine transaminase (ALT) activity and increased mitochondrial respiration state 3 rates [90].

Administration of naringenin (3% wt/wt) for 12 weeks in high-fat/high-cholesterol (HFHC) fed Ldlr^−/−^ mice increased hepatic fatty acid oxidation and attenuation of fatty acid synthesis (Table 8) [91]. Plasma glucose, insulin, total cholesterol, triglyceride, VLDL and LDLD levels were significantly reduced with naringenin treatment. Additionally, naringenin attenuated ApoB100 section by 80% in the HFHC-induced mice. Hepatic *Srebf1c* and *Acox1* mRNA levels were reduced, while *Fgf21, Pgc1a,* and *Cpt1a* mRNA levels were increased with naringenin treatment. In addition, hepatic pro-inflammatory cytokine (*Tnfa, Il1b, Ccl2,* and *Ccl3*) mRNA levels were reduced [91]. 

Treatment with naringenin (50 and 100 mg/kg/day) for 14 days had no effect on blood glucose levels of diabetic C57BL/6J mice fed a high-fat diet in a study by Yoshida, et al., (2014) [92]. However, naringenin significantly repressed MCP-1 levels in adipose tissue and suppressed overall macrophage infiltration [92]. These data indicate a potential of naringenin to prevent macrophage infiltration into adipose tissues and, therefore, prevent the inflammatory responses contributing to insulin resistance and diabetes (Table 8). 

Naringenin treatment (25 mg/kg p.o.) for 2 h in albino Wistar rats fed a high-fat diet significantly reduced the maltose- and sucrose-loaded glycemic response by 50.64% and 51.02%, respectively [72]. Moreover, naringenin significantly inhibited α-glucosidase enzymatic activity leading to delayed intestinal carbohydrate absorption and reduced blood glucose levels (Table 8) [72]. 

Naringenin treatment (3% wt/wt) for 16 weeks of high fat diet-induced diabetic C57BL6/J and FGF21^−/−^ mice significantly reduced dyslipidemia and improved glucose tolerance [93]. Naringenin treatment in both HFD-C57BL6/J and HFD-FGF21^−/−^ mice significantly reduced plasma glucose and insulin levels (Table 8). Furthermore, treatment with naringenin reduced HFD-induced visceral and subcutaneous adipose tissue accumulation. Plasma leptin and TNFα levels were decreased with naringenin treatment to levels comparable to healthy non-diabetic animals. In addition, hepatic and white adipose *Pgc1a* and *Cpt1a* mRNA levels were significantly reduced, while *Pnpla2* and *Lipe* mRNA levels were increased with naringenin treatment [93].

Naringenin treatment (50 mg/kg b.w.) for 90 days in high-cholesterol (10 g cholesterol/kg and 1 g cholic acid/ kg) fed male Wistar rats significantly prevented renal failure (Table 8) [94]. Plasma and urine urea levels were significantly reduced, while creatinine clearance rates were increased with naringenin treatment. Histological parameters, white blood cells and platelets were increased with the high-cholesterol diet, and significantly reduced with naringenin treatment. Additionally, naringenin significantly decreased the lipid profile and kidney pro-oxidant inflammation marker (NTPDases, CD73, iNOS, TNF-α, IL-6 and NF-κB) mRNA levels [94]. These data indicate that naringenin treatment improves renal function and may protect against diabetic nephropathy. 

In a study by Krishnamoorthy et al., (2017), oral administration of naringenin (50 mg/kg b.w./day) for 6 weeks in high fructose-fed diabetic rats resulted in a significant increase in GLUT4 translocation in skeletal muscle [48]. This was accompanied by increased in skeletal muscle AMPK phosphorylation and increased SIRT1 and PGC-1α protein levels (Table 8) [48].

Naringenin treatment (3% wt/wt) for 12 weeks in high-fat/high-cholesterol (24% fat and 0.2% cholesterol caloric intake) fed Ldlr^−/−^ mice significantly increased energy expenditure and hepatic fatty acid oxidation [95]. Naringenin treatment significantly decreased body weight, epididymal fat accumulation, and visceral and subcutaneous fat volume. Adipose tissue inflammation was significantly reduced with decreased mRNA levels of pro-inflammatory genes, *Tnfa*, *Ccl2* and *Ccl3*. Naringenin also significantly reduced fasting serum insulin and glucose levels comparable to healthy non-diabetic animals. Skeletal muscle and hepatic triglyceride accumulation was reduced, while hepatic fatty oxidation gene (*Pgc1a* and *Cpt1a*) expression was increased with naringenin treatment (Table 8) [95]. 

Overall, these studies indicate that naringenin and naringin treatment of diet-induced diabetic animals resulted in restoration of serum glucose and lipid levels, increased liver and skeletal muscle fatty acid oxidation and increased skeletal muscle GLUT4 translocation (Table 8). 

### 2.6. Biological Effects of Naringenin: Human Studies

There are only two epidemiological studies examining the association between naringenin intake and health outcomes (Table 9). The intake of a variety of flavonoids, including naringenin (5.0 mg/kg/day), was associated with reduced risk of mortality from ischemic heart disease, reduced incidence of cerebrovascular disease, specific cancers and asthma [96]. Importantly, the intake of naringenin (5.0 mg/kg/day) had a tendency to reduce the risk of T2DM (Table 9) [96]. In another study, Farook, et al. (2015) measured blood levels of various flavones and flavanones including naringenin and found that the presence of naringenin (273.08 mg/mL) was inversely correlated with obesity [97]. Additionally, blood naringenin levels were negatively correlated with systolic blood pressure, diastolic blood pressure, serum triglyceride levels, waist circumference, and insulin resistance, while positively correlated with serum HDL levels (Table 9) [97]. An association was observed between the intake of citrus flavonoid-containing foods and lipid-lowering and insulin-sensitizing properties. 

In a clinical study, administration of naringin (400 mg/capsule/day) for 8 weeks in hypercholesterolemic individuals resulted in reduced plasma total cholesterol and LDL cholesterol concentrations, while HDL and triglyceride concentrations were unaffected (Table 9) [98]. In addition, apoB levels were significantly reduced with naringin treatment. Erythrocyte SOD and CAT activities were significantly increased, while GSH-Px activity and plasma TBARS levels were reduced with naringin treatment [98]. In another study, administration of a citrus polyphenol extract (900 mg/kg), containing naringin for 12 weeks in overweight individuals, resulted in significantly reduced body weight and plasma glucose levels compared to the control placebo overweight group (Table 9) [99]. These data show that administration of naringin reduced blood lipid and glucose levels, increase anti-oxidant enzyme activities and reduced body weight. Unfortunately, no measurements of plasma naringin or its metabolite levels were performed. These studies indicate that naringenin administration may have potent anti-diabetic effects in humans and further studies are required.

## 3. Conclusions

Overall, all available in vitro and in vivo animal studies examining the effects of naringenin indicate that it can reduce glucose adsorption by the intestinal brush border, reduce renal glucose reabsorption, and increase glucose uptake and utilization by muscle and fat tissues. In hepatocytes, naringenin treatment reduces triglyceride production and gluconeogenesis resulting in the attenuation of hyperglycemia and hyperlipidemia. Evidence also shows a productive effect of naringenin in the pancreas, salvaging the remaining β-cells and increasing their glucose-sensing capacity and response to glucose. The limited human studies indicate that naringenin administration can result in significant (micromolar range) plasma naringenin levels. Furthermore, the limited epidemiologic evidence indicate anti-diabetic effects of naringenin. However, more research must be conducted to fully understand the effects of naringenin in specific tissues of the body, particularly skeletal muscle, adipose tissue, liver and pancreatic β-cells. Overall, the health benefits of naringenin are widespread, and the low toxicity of the molecule makes it a prime candidate for medicinal use against insulin resistance and T2DM. Clearly, more human studies are required to explore the anti-diabetic potential of naringenin in a clinical setting, in individuals suffering from insulin resistance, obesity and T2DM.

## Figures and Tables

**Table 1:** Effects of naringenin: in vitro skeletal muscle cell studies.

Cell	Naringenin Concentration/Duration	Effect	Reference
L6 muscle cells	10–75 µM, 2 h	↑ Glucose uptake ↑ Phospho-AMPK	[46]
Primary porcine myotubes	*Sambucus nigra* flower (elderflower); Naringenin 0.1–10 µM, 1 h	↑ Glucose uptake ↓ ROS levels	[47]
L6 myotubes insulin resistance induced by palmitate (750 µM)	50 and 75 µM, 16 h	↑ Glucose uptake ↑ GLUT4 translocation ↑ Phospho-AMPK ↑ SIRT1 ↑ PGC-1α	[48]

5′ AMP-activated protein kinase (AMPK); reactive oxygen species (ROS); Glucose transporter 4 (GLUT4); Sirtuin 1 (SIRT1); Peroxisome proliferator-activated receptor gamma coactivator 1-alpha (PGC-1α).

**Table 2:** Effects of naringenin: in vitro adipocyte studies.

Cell	Naringenin Concentration/Duration	Effect	Reference
3T3-L1 preadipocytes	5–100 µM, 48 h	↓ Adipocyte proliferation ↑ LDH release	[51]
3T3-L1 adipocytes	100 µM, 30 min	↓ TNF-α FFA secretion ↓ IκB-α degradation ↓ Phospho-ERK protein expression ↑ Perilipin mRNA ↑ PDE3B mRNA	[52]
3T3-L1 adipocytes and mature human adipocytes	20 µM, 2 min	↓ Insulin-stimulated glucose uptake ↓ GLUT4 recruitment	[53]
3T3-L1 adipocytes	10, 50 and 100 µM, 0.5–3 h	↓ Inflammation ↓ TLR2 expression ↓ TNF-α ↓ MCP-1	[54]
3T3-L1 differentiating and mature adipocytes	0–50 µM, 0–120 h (Pre-adipocytes) and 0–24 h (Mature)	↓ Adipogenesis ↓ Lipid accumulation ↓ aP2, PPARγ, STAT5A and adiponectin protein ↓ IRS-1 (Y896) ↓ Adiponectin	[55]
Human white adipocytes	8 µM, 7 to 14 days	↑ GLUT4 mRNA ↑ Adiponectin mRNA ↑ UCP1, ATGL, CPT1β, PGC-1α and PGC-1β mRNA ↑ Oxygen consumption rate	[56]

Lactate dehydrogenase (LDH); Tumor necrosis factor alpha (TNFα); Nuclear factor of kappa light polypeptide gene enhancer in B-cells inhibitor, alpha (IκBα); Extracellular signal-regulated kinases (ERK); Phosphodiesterase 3B (PDE3B); Toll-like receptor 2 (TLR2); Monocyte chemoattractant protein-1 (MCP-1); Peroxisome proliferator-activated receptor gamma (PPARγ); Signal transducer and activator of transcription 5A (STAT5A); Insulin receptor substrate-1 (IRS-1); Uncoupling protein 1 (UCP1); Adipose triglyceride lipase (ATGL); Carnitine palmitoyltransferase 1β (CPT1β).

**Table 3:** Effects of naringenin: in vitro hepatocyte studies.

Cell	Naringenin Concentration/Duration	Effect	Reference
HepG2 human hepatoma cells	10–200 µM, 24 h	↓ Apo B secretion ↓ ACAT2 mRNA ↓ MTP protein and mRNA ↑ LDL receptor mRNA ↑ LDL uptake ↑ LDL degradation	[58]
HepG2 human hepatoma cells	200 µM, 6 h	↓ Apolipoprotein B secretion ↑ SREBP-1 and LDLr expression ↑ PI3K activity	[59]
HepG2 human hepatoma cells	0–200 µM, 60 min	↓ Apo B secretion ↑ ERK activity ↓ Microsomal triglyceride transfer protein	[60]
Hepatoma (Fao) cells	6–100 µM, 6 h	↓ Glucose production ↓ Cellular ATP levels	[61]
Huh7 hepatocytes and Lewis rat primary hepatocytes	0–380 µM, 16–24 h	↓ Triglyceride production ↑ Fatty acid oxidation ↓Trap220/Drip-2 and LBD ↓ LXRα response element ↑ mRNA of CYP4A11, ACOX, UCP1 and ApoAI	[62]
Wistar rat hepatocytes	300 µM, 30–50 min	↓ Glucose production ↓ Gluconeogenesis ↓ Pyruvate transport	[63]

Apolipoprotein B (Apo B); Acetyl-CoA acetyltransferase 2 (ACAT2); Microsomal triglyceride transfer protein (MTP); Sterol regulatory element-binding protein-1 (SREBP-1); Phosphoinositide 3-kinase (PI3K); Cytochrome P450 family 4 A member 11 (CYP4A11); Peroxisomal acyl-CoA oxidase (ACOX).

**Table 4:** Effects of naringenin: in vitro beta cell studies.

Cell	Naringenin Concentration/Duration	Effect	Reference
INS-1E cells	100–1000 µM, 1 and 72 h	↑ Glucose-stimulated insulin secretion ↑ *Glut2, Gck, Ins1/2, Beta2* and *Pdx1* mRNA ↑ *Akt1, Akt2, Irs1, Bcl2* and *Hsp70/90* mRNA ↓ *Bax, Casp3*, and *Acc1* mRNA	[64]

Glucose transporter 2 (Glut2); Glucokinase (Gck); Insulin 1/2 (Ins1/2); β2 adrenergic receptor agonists (Beta2); Insulin promoter factor 1 (Pdx1); Protein kinase B (Akt); B-cell lymphoma 2 (Bcl2); Heat shock protein 70/90 (Hsp70/90); Bcl2 associated X (Bax); Caspase 3 (Casp3); Acetyl-CoA carboxylase 1 (Acc1).

**Table 5:** Evidence of anti-diabetic effects of naringenin: in vivo streptozotocin (STZ)-induced diabetes animal studies.

Streptozotocin (STZ)-Induced Diabetes Animal Models				
Animal	Naringenin Concentration/Duration	Blood Measures	Other Measures	Reference
Neonatal Wistar rats and New Zealand White rabbits	0.1–1000 mM, 1–2 min	No effect	↓ Intestinal BBMV glucose uptake ↓ renal BBMV glucose uptake	[65]
Wistar rats	5 and 50 mg/kg b.w., 5 days	↓ Glucose levels ↓ Total Cholesterol ↓ Triglycerides ↑ HDL levels	No effect	[66]
Wistar rats	30 mg/kg b.w., 21 days	↓ Glucose levels ↑ Insulin levels ↑ Vitamin E levels	↑ Kidney and liver hexokinase activity ↓ Liver and kidney G6Pase and F16BPase ↓ Glycoprotein levels	[67]
Male BALB/cA mice	0.5–2% dietary intake, 10 weeks	↓ Glucose levels ↑ Insulin levels	↓ Blood urea nitrogen levels ↓ Kidney NF-κB p65 ↓ Renal TNFα ↓ Renal IL-1β ↓ Renal IL-6 ↓ Renal CCP-1	[68]
Wistar albino rats	25, 50 and 100 mg/kg. b.w/day, 28 days	↓ Glucose levels ↑ Insulin levels ↓ Triglyceride, TAG, LDL and NEFA levels	↑ Liver and kidney PPARγ ↑ HSP-27 and HSP-72 protein ↓ Pancreatic, liver and kidney NF-κB protein ↓ Liver TNFα ↓ Renal IL-6	[69]
Albino Wistar rats	50 mg/kg b.w./day, 21 days	↓ Fasting-glucose levels ↓ HbA1c ↑ Insulin levels	↑ SOD ↑ Catalase ↑ GSH peroxidase ↑ Glutathione-S-transferase activity	[70]
Male albino rats	50 mg/kg b.w., 4 weeks	↓ Glucose levels ↑ Insulin levels ↓ HbA1c levels	↓ Lipid peroxide and NO levels ↑ Vitamin C, vitamin E and GSH levels ↓ TNF-α and IL-6 levels	[71]
Albino Wistar rats	25 mg/kg p.o., 2 h	↓ Glucose in response to maltose and sucrose load	↓ α-glucosidase activity	[72]
Male Wistar rats	20, 50, and 100 mg/kg p.o, 8 weeks	↓ Glucose levels	↓ Hyperalgesia ↑ SOD ↑ Body weight	[73]
Male Wistar rats	50 mg/kg b.w., 30 days	↓ Glucose levels	↓ Oxidative stress ↓ Liver ROS ↓ Liver lipid peroxidation ↓ Bax and Bcl-2 mRNA and protein	[74]
Male Wistar rats	25 and 50 mg/kg b.w./day, 5 weeks	↓ Glucose levels ↑ Insulin levels ↓ TNF-α, IL-1β and IL-6 levels	↑ Sciatic IGF expression ↑ Sciatic NGF expression ↓ TBARS, GSH levels ↑ SOD and catalase activity ↓ Sciatic histology	[75]
Sprague-Dawley rats	50 and 100 mg/kg/day, 6 weeks	↑ Glucose tolerance ↓ Glucose levels ↓ Lipid levels	↓ ICAM-1 ↓ Malonaldehyde levels	[76]
Sprague–Dawley rats	50 mg/kg b.w./day, 6 weeks	↓ Glucose levels	↓ Urinary protein levels ↓ Kidney index ↑ Creatinine clearance ratio ↓ ECM deposition ↑ let-7a signaling ↓ TGF-β1 and TGFBR1 mRNA and protein ↓ Smad2 and smad7 mRNA and protein	[77]
Male Wistar rats	50 and 100 mg/kg b.w., 10 weeks	↓ Glucose levels ↓ Total cholesterol levels	↑ Kidney SOD ↑ Kidney Catalase ↑ Kidney GSH ↓ Renal IL-1	[78]
Male Wistar rats	50 mg/kg b.w./day, 5 weeks	↓ Glucose levels ↑ Insulin levels	↓ Retinal TBAR levels ↓ GSH levels ↑ BDNF and TrkB levels ↑ Synaptophysin levels ↑ Bcl-2 protein ↓ Bax and Caspase-3 protein	[79]
Male albino rats	100 mg/kg b.w./day, 4 weeks	↑ Insulin levels ↑ Lipid profile	↑ GLUT4 mRNA ↓ Liver G6Pase activity	[80]
Male albino rats	100 mg/kg/day, 15 days	↓ Glucose levels ↑ Lipid levels	↑ GLUT4 activity ↑ PPARγ in the pancreas	[81]

Brush border membrane vesicle (BBMV); High-density lipoproteins (HDL); Glucose 6-phosphatase (G6Pase); Fructose 1,6-bisphosphatase (F16BPase); Nuclear factor kappa-light-chain-enhancer of activated B cells (NF-κB); Interleukin 1β (IL-1β); Cytochrome c peroxidase-1 (CCP-1); Triacylglycerol (TAG); Non-esterified fatty acids (NEFA); Superoxide dismutase (SOD); Glutathione (GSH); Nitric oxide (NO); Hemoglobin A1c (HbA1c); Insulin-like growth factor (IGF); Nerve growth factor (NGF); Thiobarbituric acid reactive substances (TBARS); Intercellular adhesion molecule 1 (ICAM-1); Extracellular matrix (ECM); Transforming growth factor beta 1 (TGF-β1); Transforming growth factor beta receptor 1 (TGFBR1); Brain-derived neurotrophic factor (BDNF); Tropomyosin receptor kinase B (TrkB).

**Table 6:** Evidence of anti-diabetic effects of naringenin: in vivo alloxan-induced diabetes animal studies.

Alloxan-Induced Diabetes Animal Model				
Animals	Naringenin Concentration/Duration	Blood Measures	Other Measures	Reference
Swiss albino mice	50 mg/kg b.w., 7 days	↑ Immunological parameters ↓ Cholesterol levels	↑ Survival	[82]
Swiss albino mice	50 mg/kg b.w., 7 days	↓ Lipid peroxidation levels	↑ Tissue repair ↓ Liver vacuolisation	[83]

**Table 7:** Evidence of anti-diabetic effects of naringenin: in vivo genetic diabetes animal studies.

Genetically-Induced Diabetes Animal Model				
Animals	Naringenin Concentration/Duration	Blood Measures	Other Measures	Reference
C57BL/KsJ-*db*/*db* mice	0.2 g/kg diet, 5 weeks	↓ Glucose levels ↓ Lipid levels	↓ Hepatic FAS, G6Pase and PAP activity ↓ Hepatic fatty acid β-oxidation ↑ Hepatic GK mRNA ↑ GLUT4 levels ↓ GLUT2 levels	[84]
Ldlr^−/−^ mice	3% wt/wt, 8 weeks	↓ Glucose levels ↓ Lipid levels ↓ Insulin levels ↑ β-hydroxybutyrate	↑ Energy expenditure ↑ *Pgc1a* mRNA ↑ *Cpt1a* mRNA ↑ *Pnpla2* mRNA	[85]

Fatty acid synthase (FAS); Phosphatidate phosphatase (PAP); Glucokinase (GK); Carnitine palmitoyltransferase 1α (Cpt1α); Patatin like phospholipase domain containing 2 (Pnpla2).

**Table 8:** Evidence of anti-diabetic effects of naringenin: in vivo diet-induced diabetes animal studies.

Diet-Induced Diabetes Model				
Animal Model	Naringenin Concentration/Duration	Blood Measures	Other Measures	Reference
Male rabbits; high-cholesterol diet	0.05% naringin, 8 weeks	↓ LDL-cholesterol ↑ HDLD cholesterol	↑ Hepatic CoA reductase ↓ Acyl-CoA cholesterol acyltransferase activity	[86]
Ldlr^−/−^ mice; high fat diet	1–3% wt/wt, 4 weeks	↑ Hepatic glucose tolerance	↓ Hepatic cholesterol ↓ Muscle VLDL fatty acids ↑ Hepatic fatty acid oxidation	[87]
Ldlr^−/−^ mice; high fat diet	3% wt/wt, 6 months	↓ Triglyceride levels ↓ Cholesterol levels	↓ Aortic plaque deposits ↓ Atherosclerosis ↓ Liver triglyceride ↓ Liver cholesteryl ester mass	[38]
Wistar rats; high fructose diet	50 mg/kg b.w, 45 days	↑ Glycogen content (liver and skeletal muscle)	↑ phospho-PTK	[43]
Male Long-Evans hooded rats; high-sucrose diet	0.003, 0.006 and 0.012% dietary consumption, 6 weeks	↓ Triglyceride levels ↓ Cholesterol levels	↓ Hepatic cholesterol ↓ Adipose triglyceride levels ↑ PPARα protein ↑ CPT-1 and UCP2 protein	[88]
C57BL/6 mice; high-fat diet	0.2 g/kg diet, 20 weeks	No effect	↓ MAPK signaling ↑ IRS1 ↑ Fatty acid oxidation ↑ AMPK activation	[89]
BALB/cA mice; high fat-fed diet	0.5–2% dietary intake, 4 months	↓ Glucose levels ↑ Insulin levels	↓ TNF-α ↓ MCP-1 ↓ TLR2 in adipose tissues	[54]
Wistar rats; high-fat-high-cholesterol diet	100 mg/kg/day, 16 weeks	↓ Glucose levels ↓ Insulin levels ↓ Cholesterol levels ↓ Triglyceride levels ↓ NEFA levels ↓ AST and ALT activity	↓ Inflammatory cell infiltration ↓ Collagen deposition ↑ Mitochondrial respiration state 3 rates	[90]
Ldlr^−/−^ mice	3% naringenin, 12 weeks	↓ Glucose levels ↓ Insulin levels ↓ Lipid levels	↓ ApoB100 secretion ↓ *Srebf1c* and *Acox1* mRNA ↑ *Fgf21, Pgc1a,* and *Cpt1a* mRNA ↓ *Tnfa, Il1b, Ccl2,* and *Ccl3* mRNA	[91]
C57BL/6J; high fat diet	100 mg/kg/day, 14 days	No effect	↓ MCP-1 ↓ Macrophage infiltration in adipose tissues	[92]
Albino Wistar rats; high fat diet	25 mg/kg p.o., 2 h	↓ Glucose levels	↓ α-glucosidase activity	[72]
C57BL6/J and FGF21^−/−^ mice; high-fat diet	3% wt/wt, 4 weeks	↓ Glucose levels ↑ Insulin levels ↓ Leptin levels ↓ TNFα levels	↓ Obesity ↓ Adipose tissue volume ↓ *Pgc1a* and *Cpt1a* mRNA ↑ *Pnpla2* and *Lipe* mRNA	[93]
Male Wistar rats; high cholesterol diet	50 mg/kg b.w./day, 90 days	↓ Lipid levels	↓ Renal urea levels ↑ Creatinine clearance rate ↓ Renal WBCs and platelets levels ↓ NTPDases, CD73, iNOS, TNF-α, IL-6 and NF-κB mRNA	[94]
Albino Wistar rats; high fructose diet	50 mg/kg b.w./day, 6 weeks	No effect	↑ Skeletal muscle GLUT4 translocation ↑ Phospho-AMPK ↑ SIRT1 ↑ PGC-1α	[48]
Ldlr^−/−^ mice; high-fat/high-cholesterol diet	3% wt/wt, 12 weeks	↓ Fasting glucose levels ↓ Fasting insulin levels	↓ Hepatic lipid levels ↓ Skeletal muscle lipid levels ↑ *Pgc1a* and *Cpt1a* mRNA	[95]

Very-low-density lipoproteins (VLDL); Protein tyrosine-kinase (PTK); Mitogen-activated protein kinase (MAPK); Aspartate transaminase (AST); Alanine transaminase (ALT); Sterol regulatory element binding protein 1c (Srebf1c); Fibroblast growth factor 21 (Fgf21); Lipase E (Lipe); White blood cells (WBC); Nucleoside triphosphate diphosphohydrolase (NTPDases); Cluster of differentiation 73 (CD73); Nitric oxide synthase (iNOS).

**Table 9:** Effects of naringenin: Human studies.

Naringenin Concentration	Blood Measures	Other Measures	Reference
3.7–5.0 mg/kg/day	↓ Cholesterol levels	↓ T2DM risk ↓ Asthma ↓ Ischemic heart disease ↓ Mortality	[96]
273.08 mg/mL	↓ Triglyceride levels ↑ HDL levels	↓ Obesity ↓ Systolic and diastolic blood pressure	[97]
400 mg/capsule/day	↓ Cholesterol levels ↓ LDL levels ↓ ApoB levels ↓ TBARS levels	↑ SOD activity ↑ CAT activity ↓ GSH-Px activity	[98]
900 mg/kg	↓ Glucose levels	↓ Body weight	[99]

Type 2 diabetic mellitus (T2DM); Glutathione peroxidase (GSH-Px).
